# KMUP-1 Ameliorates Ischemia-Induced Cardiomyocyte Apoptosis through the NO–cGMP–MAPK Signaling Pathways

**DOI:** 10.3390/molecules24071376

**Published:** 2019-04-08

**Authors:** Meng-Luen Lee, Erna Sulistyowati, Jong-Hau Hsu, Bo-Yau Huang, Zen-Kong Dai, Bin-Nan Wu, Yu-Ying Chao, Jwu-Lai Yeh

**Affiliations:** 1 Division of Pediatric Cardiology, Department of Pediatrics, Changhua Christian Children’s Hospital, Changhua 50050, Taiwan; ferdielee@yahoo.com; 2 School of Medicine, College of Medicine, Kaohsiung Medical University, Kaohsiung 807, Taiwan; 3 Graduate Institute of Medicine, College of Medicine, Kaohsiung Medical University, Kaohsiung 80708, Taiwan; dr_erna@unisma.ac.id (E.S.); jhh936@yahoo.com.tw (J.-H.H.); peter71129@gmail.com (B.-Y.H.); zenkong@kmu.edu.tw (Z.-K.D.); binnan@kmu.edu.tw (B.-N.W.); 4 Faculty of Medicine, University of Islam Malang, Malang city, East Java Province 65145, Indonesia; 5 Department of Pediatrics, College of Medicine, Kaohsiung Medical University, Kaohsiung 80708, Taiwan; 6 Department of Pediatrics, Kaohsiung Medical University Hospital, Kaohsiung 807, Taiwan; 7 Department of Pharmacology, College of Medicine, Kaohsiung Medical University, Kaohsiung 80708, Taiwan; 8 Department of Public Health, College of Health Sciences, Kaohsiung Medical University, Kaohsiung 80708, Taiwan; yuyich@kmu.edu.tw; 9 Department of Medical Research, Kaohsiung Medical University Hospital, Kaohsiung 80708, Taiwan; 10 Department of Marine Biotechnology and Resources, National Sun Yat-sen University, Kaohsiung 80424, Taiwan

**Keywords:** KMUP-1, cardiomyocyte apoptosis, hypoxia, nitric oxide

## Abstract

To test whether KMUP-1 (7-[2-[4-(2-chlorophenyl) piperazinyl]ethyl]-1,3-dimethylxanthine) prevents myocardial ischemia-induced apoptosis, we examined KMUP-1-treated H9c2 cells culture. Recent attention has focused on the activation of nitric oxide (NO)-guanosine 3’, 5’cyclic monophosphate (cGMP)-protein kinase G (PKG) signaling pathway triggered by mitogen-activated protein kinase (MAPK) family, including extracellular-signal regulated kinase 1/2 (ERK1/2), c-Jun N-terminal kinase (JNK), and p38 in the mechanism of cardiac protection during ischemia-induced cell-death. We propose that KMUP-1 inhibits ischemia-induced apoptosis in H9c2 cells culture through these pathways. Cell viability was assessed using MTT (3-(4,5-dimethylthiazol-2-yl)-2,5-diphenyltetrazolium bromide) assay and apoptotic evaluation was conducted using DNA ladder assay and Hoechst 33342 staining. The level of intracellular calcium was detected using-Fura2-acetoxymethyl (Fura2-AM) staining, and mitochondrial calcium with Rhod 2-acetoxymethyl (Rhod 2-AM) staining under fluorescence microscopic observation. The expression of endothelium NO synthase (eNOS), inducible NO synthase (iNOS), soluble guanylate cyclase α1 (sGCα1), PKG, Bcl-2/Bax ratio, ERK1/2, p38, and JNK proteins were measured by Western blotting assay. KMUP-1 pretreatment improved cell viability and inhibited ischemia-induced apoptosis of H9c2 cells. Calcium overload both in the intracellular and mitochondrial sites was attenuated by KMUP-1 pretreatment. Moreover, KMUP-1 reduced intracellular reactive oxygen species (ROS), increased plasma NOx (nitrite and nitrate) level, and the expression of eNOS. Otherwise, the iNOS expression was downregulated. KMUP-1 pretreatment upregulated the expression of sGCα1 and PKG protein. The ratio of Bcl-2/Bax expression was increased by the elevated level of Bcl2 and decreased level of Bax. In comparison with the ischemia group, KMUP-1 pretreatment groups reduced the expression of phosphorylated extracellular signal-regulated kinases ERK1/2, p-p38, and p-JNK as well. Therefore, KMUP-1 inhibits myocardial ischemia-induced apoptosis by restoration of cellular calcium influx through the mechanism of NO-cGMP-MAPK pathways.

## 1. Introduction

Currently, ischemic heart disease is the leading cause of global cardiovascular-related mortality with some 15.2 million deaths in 2016 [1,2]. The prediction made by the World Health Organization shows that ischemic heart disease will be the most common cause of death in the world until 2030 while the cause of disability will rise from sixth to third position [1]. A multitude of recent studies suggests that ischemia injury leads to undergoing cardiomyocyte apoptosis and results in myocardial cell loss. Consequently, it develops cardiac dysfunction, ventricular remodeling, and contributes to deterioration with heart failure [3]. More recent evidence has indicated that the inhibition of apoptosis results in reducing myocardial damage, decreasing cardiomyocyte removal, and ventricular contractile function improvement [4]. 

The first concept of apoptosis was proposed by Kerr et al. (1972); cell shrinkage, increased cytoplasmic density, chromatin condensation, nuclear DNA degradation, intracellular membranes surrounding the DNA fragment, and formation of the apoptosis body are characterized by an apoptotic cell [5]. Recently, three principle apoptosis signaling pathways have become known including the mitochondrial signaling pathway, the death receptor signaling pathway, and the endoplasmic reticulum signaling pathway. Notwithstanding, many researchers have assumed that this mechanism remains unclear [5,6,7,8]. Interestingly, the signaling cascades of the mitogen-activated protein kinase (MAPK) pathway and c-Jun N-terminal kinase (JNK) have been involved in hypoxia-induced H9c2 embryonic rat heart-derived cell line apoptosis [9,10]. Furthermore, NO and cGMP pathway takes an important role in the modulation of cardiac protection during ischemia. NO increases intracellular cGMP level via the activation of sGC or particulate guanylate cyclase (pGC) to inhibit cardiac myocytes apoptosis [11]. 

**Figure 1:**
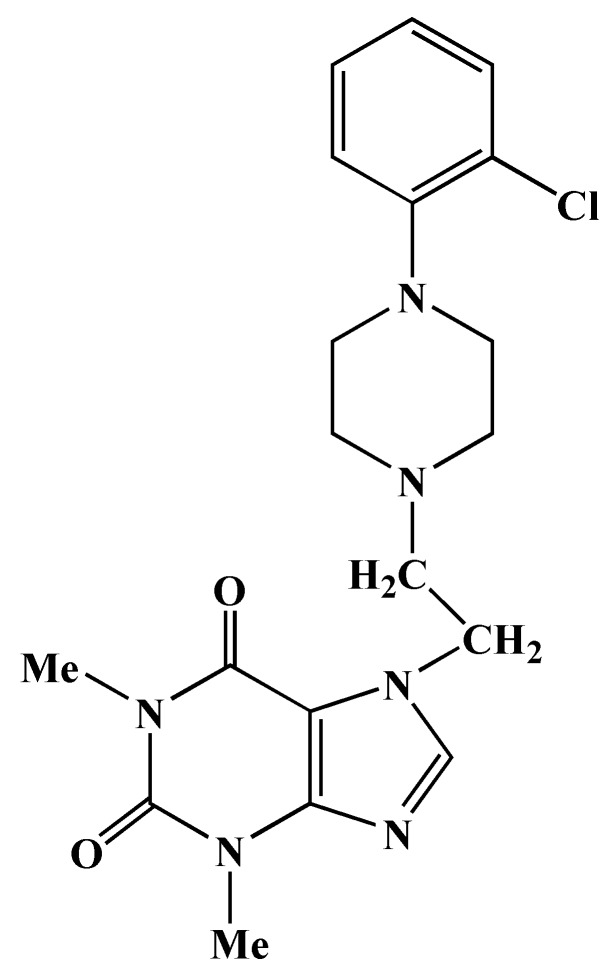
Chemical structure of KMUP-1 (7-[2-[4-(2-chlorophenyl) piperazinyl]ethyl]-1,3-dimethylxanthine).

A unique xanthine and piperazine derivative developed by our group, 7-[2-[4-(2-chlorophenyl) piperazinyl]ethyl]-1,3-dimethylxanthine, known as KMUP-1 (Figure 1), was demonstrated to play a critical function in the hypoxia state [12,13]. However, our recent studies showed that sGC activation, an increase of cGMP, and phosphodiesterases inhibition resulted from KMUP-1 treatment. Consequently, it leads to smooth muscle relaxation in aorta [14], trachea [15], and corporeal cavernous [16]. In this study, we hypothesized that KMUP-1 protects cardiac myocytes apoptosis through NO-cGMP-PKG signaling pathways by activating the MAPK family, including ERK1/2, JNK, and p38. To test this, we generated KMUP-1 pretreatment in the hypoxia-induced H9c2 cell, a cell line with a myogenic potential derived from embryonic rat hearts following apoptosis evaluation together with certain protein assays to determine the pathways.

## 2. Results

### 2.1. Effect of KMUP-1 on Hypoxia-Induced Cytotoxicity

**Figure 2:**
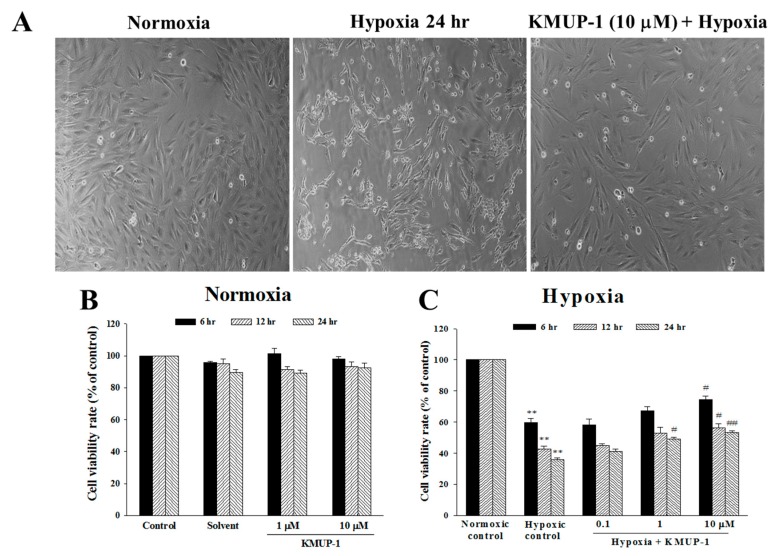
Effect of hypoxia on H9c2 cell viability. Cells were cultured in normoxia or hypoxia for 24 h. (**A**) Phase-contrast photomicrographs; normoxia, hypoxia, and KMUP-1 (10 µM) pretreatment. The cell viability rate of H9c2 cell under (**B**) normoxic condition, and (**C**) hypoxic condition. The observation time was at 6, 12, and 24 h. All values were expressed as mean ± SEM (standard error of the mean), *n* = 3. ** *p* < 0.01 vs. normoxia, # *p* < 0.05 vs. hypoxia, ## *p* < 0.01 vs. hypoxia.

To examine the effect of hypoxia exposure on cell death in H9c2 cell, we characterized its morphological appearance (Figure 2A). H9c2 embryonic rat heart-derived cell line treated with hypoxia for 24 h lost adherence and floated in the medium. Pretreatment the cardiomyocytes with 10 μM of KMUP-1 reduced cell death during hypoxia (Figure 2A). To identify whether hypoxia causes cell death, we generated the MTT assay. We made a different exposure; normoxia and hypoxia, we then compared the cell viability rate between these two groups. As shown in Figure 2B, under normoxia environment, no differences are denoted on cell viability rate among control, solvent, and KMUP-1 (1 and 10 μM) pretreatment groups. As presented in Figure 2C, under hypoxia stimulation, the H9c2 cell viability was significantly decreased (*p* < 0.01). By KMUP-1 (1 and 10 μM) pretreatment, the cell viability was significantly increased in hypoxic H9c2 cell (*p* < 0.05). These both mean that KMUP-1 pretreatment causes no cytotoxicity both in the normoxic environment and in hypoxic stimulation. 

### 2.2. KMUP-1 Prevents DNA Fragmentation in Hypoxic Cardiomyocytes

**Figure 3:**
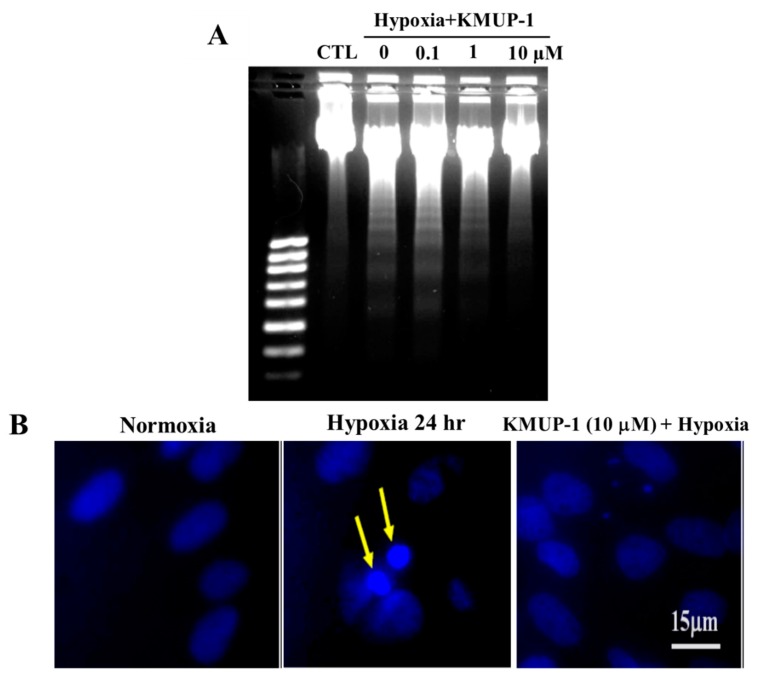
Apoptotic evaluation of H9c2 cardiomyoblasts. (**A**) DNA ladder assay of H9c2 cardiomyoblasts; normoxia (lane 1; CTL), hypoxia (lane 2), hypoxia with 0.1 μM KMUP-1 (lane 3), hypoxia with 1 μM KMUP-1 (lane 4), and hypoxia with 10 μM KMUP-1 (lane 5). (**B**) The effect of 10 μM KMUP-1 on hypoxic H9c2 cardiomyoblasts (Hoechst 33342 staining, original magnification × 400). The condensed and fragmented H9c2 cells’ nuclei and apoptotic bodies (arrows) are seen in hypoxic-H9c2 cell, but not in the normoxia and KMUP-1 treatment.

To analyze the genomic DNA, H9c2 embryonic rat heart-derived cell line was examined on DNA laddering assay with agarose gel electrophoresis. The internucleosomal DNA fragmentation, a characteristic of apoptosis could be reversed when KMUP-1 (0.1, 1, and 10 μM) was added to the culture media without sera (Figure 3A). These data demonstrate that KMUP-1 prevents apoptosis in hypoxic H9c2 cell, indicating that KMUP-1 has an anti-apoptotic role. The H9c2 cells’ dying nuclei were visualized with Hoechst 33342 staining and observed under fluorescence microscopy (Figure 3B). The fluorescence images clearly show DNA fragmentation (yellow arrows) in the hypoxic H9c2 cell. Aside of that, the nuclei of KMUP-1-treated H9c2 cells remain intact.

### 2.3. KMUP-1 Diminishes Intracellular Calcium Concentration of Hypoxic Cardiomyocytes

**Figure 4:**
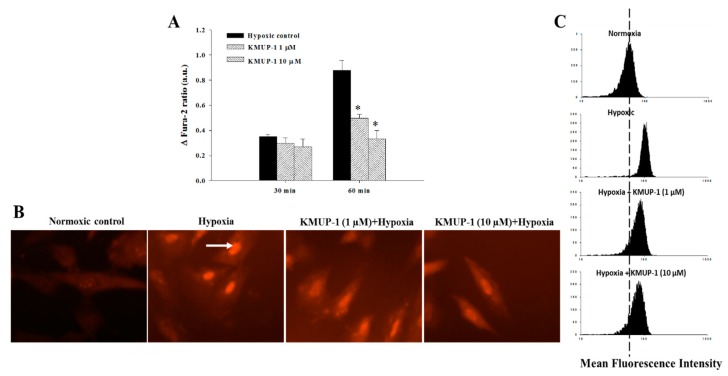
Evaluation of Ca^2+^ concentration [Ca^2+^] in H9c2 cardiomyoblasts. (**A**) A representative of intracellular Ca^2+^ concentration ([Ca^2+^]i) fluorescence intensity in H9c2 cells after 24 h KMUP-1 pretreatment and then incubated with hypoxia for 30 min and 60 min, respectively. Fura-2-AM labeling in H9c2 cells shows that KMUP-1 pretreatment (1 and 10 µM) groups have lower [Ca^2+^]i compared with both hypoxic control groups. (**B**) Mitochondrial Ca^2+^ concentration ([Ca^2+^]m) imaging with Rhod 2-AM. Fluorescence evaluation shows higher [Ca^2+^]m in hypoxia group (white arrow). (**C**) Intracellular oxidant stress evaluation with 2’,7’-dichlorofluorescin diacetate (DCFH-DA) labeling in H9c2 cells to evaluate hydroxyl generation (H_2_O_2_). Fluorescence photomicrograph shows an elevation of H_2_O_2_ generation in hypoxia group compared with normoxia and KMUP-1 (1 and 10 μM) pretreatment groups. All values were expressed as mean ± SEM, *n* = 6, * *p* < 0.05.

To determine the intracellular calcium concentration ([Ca^2+^]i) of H9c2 cell, we measured the ratio of Fura-2 AM staining (arbitrary units/a.u). We then observed the cell at two periods of time, 30 and 60 min, respectively. As shown in Figure 4A, at both of these two periods of observation, the [Ca^2+^]i is higher in the hypoxia groups. Aside of that, in the group receiving KMUP-1 (1 and 10 μM) shows a significant [Ca^2+^]i reduction (*p* < 0.05). These results suggest that KMUP-1 pretreatment inhibits the elevation of [Ca^2+^]i in cardiomyocytes under hypoxic stimulation. The mitochondrial calcium concentration ([Ca^2+^]m) was recorded in the same cell using a dual dye loading of Rhod 2-AM. As shown in Figure 4B, hypoxia caused [Ca^2+^]m in H9c2 cell to be increased (white arrow) and by KMUP-1 pretreatment (1 and 10 μM), [Ca^2+^]m to be decreased.

### 2.4. KMUP-1 Pretreatment Reduces Intracellular Reactive Oxygen Species (ROS) in Hypoxic Cardiomyocytes

To determine whether endogenous ROS levels of in vitro H9c2 cell differed from those of hypoxia and KMUP-1 pretreatment, we used ROS-sensitive dye DCFH-DA capable of detecting H_2_O_2_ [17,18]. As presented in Figure 4C, the hypoxic H9c2 cardiomyoblasts display an increase in cellular ROS relative to normoxic cells. KMUP-1 pretreatment significantly reduced the intracellular ROS generation of hypoxic cardiomyocytes (*p* < 0.05). These findings suggest that KMUP-1 pretreatment prevents hypoxia-induced oxidative stress in cardiomyocytes.

### 2.5. KMUP-1 Upregulates Plasma Nitric Oxide Level and eNOS Expression, but Downregulates iNOS Expression in Hypoxic Cardiomyocytes

**Figure 5:**
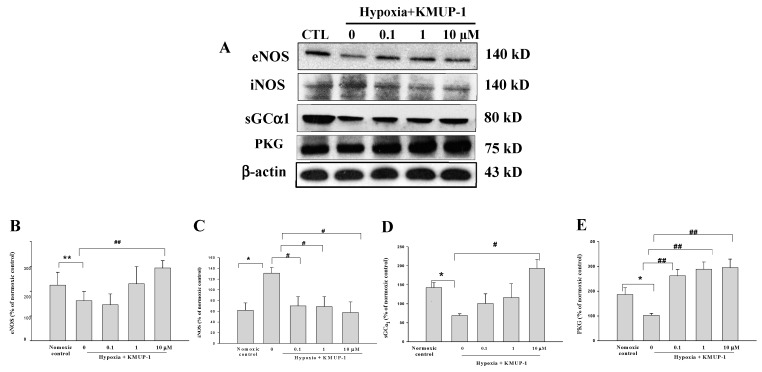
Effects of KMUP-1 on eNOS, iNOS, sGCα1, and PKG protein expressions in H9c2 cardiomyoblasts induced by hypoxia. (**A**) Western blot analyses of these proteins were performed on whole protein extracts from H9c2 cardiomyoblasts during hypoxia (24 h). Quantitative analysis protein expressions (**B**) eNOS, (**C**) iNOS (**D**) sGCα1, and (**E**) PKG. Results were presented as mean ± SEM from three independent experiments. * *p* < 0.05 vs. normoxic control, # *p* < 0.05 vs. hypoxic control ## *p* < 0.01 vs. hypoxic control.

The crucial negative modulator for cardiomyocyte apoptosis is the endogenous NO system. Therefore, we then evaluated the effects of KMUP-1 on this system. As shown in Table 1, KMUP-1 results in a significant increase of plasma nitric oxide (NO) concentration of H9c2 cell in comparison with the hypoxia group. However, as depicted in Figure 5A,B, KMUP-1 was significant in inducing expression of eNOS (*p* < 0.01), but down-regulated the expression of iNOS (*p* < 0.05) in hypoxic H9c2 cell. This findings show the NOx enhancing effect of KMUP-1 pretreatment.

### 2.6. KMUP-1 Increases the Expression of sGCα1 and PKG in Hypoxic Cardiomyocytes

To study how KMUP-1 affects myocardial cGMP and PKG, the second messenger of NO and downstream cGMP-dependent protein kinase, we investigated sGCα1 and PKG by Western blotting analyses (Figure 5D,E). Hypoxia decreased the expression of sGCα1 and PKG (*p* < 0.05) in H9c2 cell. Pretreatment with KMUP-1 significantly increased the expression of both sGCα1 (*p* < 0.05), and PKG in hypoxic H9c2 cell (*p* < 0.01). 

### 2.7. KMUP-1 Ameliorates the Ratio of Bcl-2/Bax Expression in Hypoxia-Induced H9c2 Rat Cardiomyocytes

**Figure 6:**
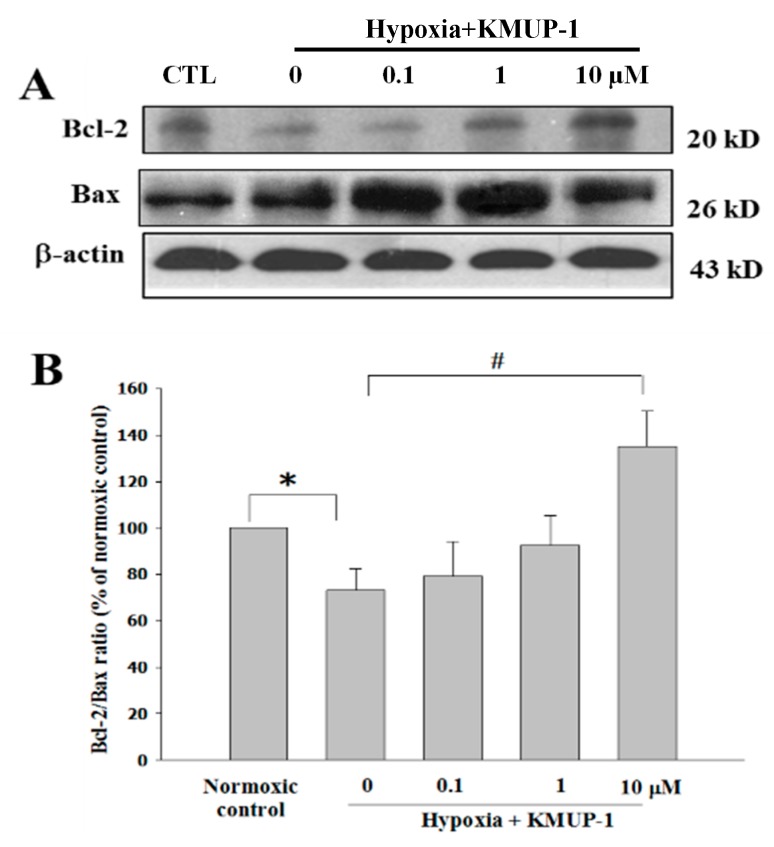
Effect of KMUP-1 on the ratio of Bcl-2/Bax proteins expression in H9c2 cardiomyoblasts induced by hypoxia. (**A**) Western blot analysis of Bcl-2/Bax proteins ratio were performed on whole protein extracts from H9c2 cardiomyoblasts cultured without sera and treated with hypoxia (24 h). (**B**) Quantitative analysis on the ratio of Bcl-2/Bax proteins expression in H9c2 cardiomyoblasts during hypoxia (24 h). Results are presented as mean ± SEM from three independent experiments. * *p* < 0.05 vs. normoxic control, # *p* < 0.05 vs. hypoxic control.

To further determine the anti-apoptotic activity of KMUP-1, we investigated both Bcl-2, an inhibitor of apoptosis, and Bax, a pro-apoptotic Bcl-2 family member. The expressions were evaluated in cultured H9c2 rat cardiomyocytes without sera induced by 24 h of hypoxia. Hypoxia-induced a decrease of Bcl-2 and an increase of Bax expressions, but these changes could be reversed by 10 μM KMUP-1 pretreatment (Figure 6). These results suggest that KMUP-1-mediated cardioprotection involves the normalization of the ratio of Bcl-2/Bax proteins expression.

### 2.8. KMUP-1 Downregulates the Expression of ERK1/2, p38, and JNK in Hypoxic Cardiomyocytes

**Figure 7:**
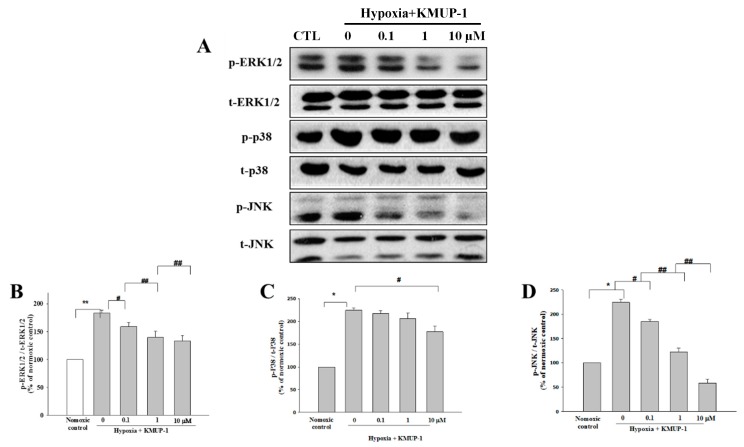
Effects of KMUP-1 on ERK1/2, p38, and JNK protein expressions in hypoxic H9c2 cell. (**A**) Western blot analyses of these proteins were performed on whole protein extracts from H9c2 cardiomyoblasts during hypoxia (24 h). Quantitative analysis protein expression of (**B**) ERK1/2, (**C**) p38, and (**D**) JNK. Results are presented as mean ± SEM from three independent experiments. * *p* < 0.05 vs. normoxic control, # *p* < 0.05 vs. hypoxic control, ## *p* < 0.01 vs. hypoxic control.

To determine whether KMUP-1 prevents the expression of phosphate ERK1/2 in hypoxic H9c2 cell, we then evaluated the blotting assay. As presented in Figure 7A,B, we found that KMUP-1 pretreatment significantly reduces its expression in comparison with the hypoxia group. To investigate further the anti-apoptotic pathway of KMUP-1 in hypoxic H9c2 cell, the expressions of p38 and JNK were assayed. As shown in Figure 7C,D, KMUP-1 pretreatment significantly declines in both their expressions in comparison with the hypoxia group. Taken together, these findings suggest that the protection of KMUP-1 might be, at least in part, in the downregulation of ERK1/2, p38, and JNK pathways.

## 3. Discussion

This present study demonstrated that KMUP-1, a novel xanthine-based derivative, could inhibit hypoxia-induced apoptosis in H9c2 cells by improving certain signaling pathways as revealed by both reduced intracellularly and mitochondrial calcium overload, activated eNOS expression, and increased NO level. Our data demonstrate that the anti-apoptotic effects of KMUP-1 regarding intracellular calcium regulation involve NO-cGMP-PKG signaling pathways and the MAPK family, including ERK1/2, JNK, and p38 in H9c2 cell in response of hypoxia-induced apoptosis.

The mechanisms contributing to the cardiomyocyte apoptosis during ischemia are complex and multifactorial. The main mediators of ischemia-induced cardiomyocyte apoptosis are increase of intracellular calcium and ROS generation. These lead to mitochondrial dysfunction as a target for downstream effects both in calcium overload and generation of ROS [19,20]. Both of these have been shown to be primary stimuli for opening the mitochondrial membrane permeability transition pore (mPTP). Upon activation of mPTP stimuli, functional breakdown and morphological disintegration of mitochondria occurs and thus initiates cell death [20,21]. In this present study, KMUP-1 inhibited intracellular calcium accumulation both in the cytosolic and mitochondrial spaces. Subsequently, ROS generation was reduced. Therefore, KMUP-1 protects hypoxia-induced cardiomyocytes apoptosis by improving cellular calcium regulation.

More emerging shreds of evidence show that the NO system plays a critical role in the modulation of cardiomyocytes apoptosis [22,23]. The contribution of NO in heart failure is elusive and theoretically disputable [24]. In this present study, we attempted to define the role of NO in the H9c2 cells cardiomyoblast apoptosis induced by hypoxia and how KMUP-1 controls the NO system in this condition. Our data demonstrated that the NO level was increased by KMUP-1 pretreatment. In addition, the expression of eNOS was also elevated. It can be suggested that the increased production of endogenous NO induced by KMUP-1 pretreatment in H9c2 cells might be primarily related to the expression of eNOS.

NO is an important gaseous signaling molecule that is synthesized by a family of NOS enzymes including inducible, neuronal, and endothelial forms [25]. eNOS is a low output-output enzyme that is constitutively expressed in H9c2 cells. NO-derived eNOS has been addressed as involving the pathophysiology of ischemic heart diseases such as myocardial infarction and myocardial ischemia-reperfusion injury [26]. A previous study reported that KMUP-1 pretreatment resulted in an increase of NO-derived eNOS, and reduced the expression of iNOS [22]. This present study was a greater extension of our previous findings about the role of KMUP-1 pretreatment in myocardial hypoxia. However, KMUP-1 increased endogenous generation of NO in ischemia-induced H9c2 cells was mainly caused by cardiac iNOS expression. Whereas, the endogenous NO production was primarily due to cardiac eNOS expression. To further explore the mechanism by which KMUP-1 attenuates myocardial cell apoptosis, we determined NO induced endothelial cell apoptosis through cGMP-independent pathways.

A growing body of literature has determined that NO-induced cGMP via the sGC receptor can block apoptosis [27]. In addition, the activation of the NO/cGMP pathway exerts a biological action to protect cardiomyocytes during ischemia [28]. Although precise mechanisms are far from clear, it is known that numerous studies suggest that cGMP-dependent protein kinase (PKG), a serine/threonine kinase activated by cGMP is involved in the inhibition of cardiomyocytes apoptosis. The increased generation of NO-sGC cascade-associated cGMP during ischemia regulates cardiac cell survival [29]. In the present study, increased NO-related cardiac cGMP and PKG activation caused by KMUP-1 pretreatment explains how it can be the braking force against ischemia-induced cardiac apoptosis. 

A large body of experimental evidence points to cGMP/PKG activation as a target for recruitment of the anti-apoptotic protein bcl-2 [29]. Bcl-2 possesses a crucial function in the regulation of mitochondrial outer membrane permeability (MOMP) and mitochondrial permeability transition pore (mPTP) activations. It is a central switch of mitochondria-dependent apoptosis [30]. Bcl-2 inhibits MOMP, while Bax protein activates MOMP. Moreover, there is a two-way crosstalk in the Ca^2+^ storage between mitochondria and the endoplasmic reticulum (ER). It is important in apoptosis associated Bcl-2 activation [31]. Bcl-2, an apoptotic inhibitor protein, facilitates Ca^2+^ leakage, allows ER Ca^2+^ unloading, and decreases mitochondrial Ca^2+^ overload. Bax, a member of the Bcl-2 family, accelerates apoptosis when it is overexpressed. Subsequently, it leads to inhibiting Ca^2+^ leakage, increases ER Ca^2+^ stores, augments ER Ca^2+^ release, increases mitochondria Ca^2+^ uptake, and ultimately accelerates mitochondria Ca^2+^ overload [30,32]. However, in the present study, KMUP-1 pretreatment increased Bcl-2 protein level and attenuated Bax protein in hypoxia-induced H9c2 cells apoptosis, and preserved the ratio of Bcl-2/Bax proteins expression. In addition, the decrease of Bcl-2/Bax expression ratio, mitochondria calcium overload, and opening of mPTP further collapsed the mitochondria membrane potential and ruptured the outer membrane of mitochondria, finally releasing pro-apoptotic molecules in the cytoplasm [33].

Mitogen-activated protein kinases (MAPKs) regulate cell survival, growth, and differentiation due to specific extracellular stimulation including hypoxia. Certain protein functions and gene expressions are altered by this pathway [9]. Abundant studies have shown that three families of MAPKs: ERK1/2, JNK, and p38 isoforms are crucial in the regulation of cardiomyocyte apoptosis. Activation of ERK1/2 during hypoxia mediates cardiomyocytes apoptosis, suggesting a pro-apoptosis role of the ERK1/2 kinases [9]. More direct evidence shows ERK1/2 stimulation contributes to apoptosis in H9c2 cells and neonatal rat cardiomyocytes [34]. In the present study, the results denote that KMUP-1 pretreatment reduced the activation of ERK1/2 in ischemia-induced cardiomyocytes apoptosis. Otherwise, p38 and JNK, the stress-activated protein kinases (SAPKs) respond predominantly to hypoxia and other environmental stress signals. Earlier studies revealed that treatment with p38 MAPK inhibitor attenuated cardiomyocytes apoptosis in vitro and in vivo [9]. The p38 possesses a detrimental role in hypoxia-induced cardiomyocytes apoptosis both in vitro and in vivo [35,36]. The inhibition of p38 preventing cardiomyocytes apoptosis is associated with an increase in the expression of the pro-survival Bcl-2 family members Bcl-2/Bcl-xl [9]. In our study, KMUP-1 pretreatment during hypoxia led to the downregulation of p38 expression. Thus, it contributes to the improvement of cell viability in hypoxia-induced H9c2 cells apoptosis. In line with this, the inhibition of JNK led to protection against I/R (ischemia reperfusion)-induced cardiomyocyte apoptosis in vitro and in vivo [18,37]. Consistent with previous reports, inactivation of JNK contributes to inhibit cardiomyocyte apoptosis caused by hypoxia [38,39]. In the present findings it was revealed that KMUP-1 pretreatment deactivated these three MAPKs protein signaling: ERK1/2, p38, and JNK expressions in hypoxia-induced H9c2 cells apoptosis. It can be proposed that KMUP-1 pretreatment inhibits cardiomyocytes apoptosis, at least in part, through MAPK pathways and warrants further investigation.

## 4. Materials and Methods

### 4.1. Chemicals and Reagents

A fluorogenic dye, 2’,7’-dichlorofluorescin diacetate (H2DCF-DA), Hoechst 33342 and Rhod-2 AM were from Molecular Probes (Eugene, Oregon, USA). Fura-2/AM, Isopropanol and MTT (3-(4,5-dimethylthiazol-2-yl)-2,5-diphenyltetrazolium bromide) were from Sigma-Aldrich (St. Louis, Missouri, USA). Anti- phospho-p38 and p38, PKG antibodies were purchased from Santa Cruz (Dallas, Texas USA); anti-eNOS, anti-iNOS, Bcl-2, Bax, and ERK 1/2 antibodies were obtained from Invitrogen (Waltham, Massachusetts, USA); anti- sGCα1 and β-actin antibodies from Sigma-Aldrich (St. Louis, Missouri, USA); anti- phospho-JNK and JNK antibodies were purchased from R&D System (McKinley Place NE, Minneapolis, USA); anti-phospho ERK 1/2 antibody was from Cell Signaling (Danvers, Massachusetts, USA). 

### 4.2. Cell Culture and Hypoxic Stimulation

The H9c2 embryonic rat heart-derived cell line was cultured in Dulbecco’s modified Eagle’s medium (DMEM) supplemented with 10% heat-inactivated fetal bovine serum, penicillin 10,000 units/mL, streptomycin 10 mg/mL, amphotericin B 0.025 mg/mL. H9c2 cells were passaged at a 1:3 ratio and sub-cultured in 100-mm diameter tissue culture dishes for 3 days. Hypoxic stimulation of H9c2 cell was achieved by using the Water-Jacketed Incubator (Forma Scientific Inc., USA). This unique system was designed whereby the incubator chamber was fitted with an oxygen sensor and constantly monitored the chamber atmosphere and maintained a stable oxygen level between 0% and 20%. The ischemic medium was prepared by Hepes-buffered salt solution (HBSS) containing (mM): NaCl, 125; KCl, 4.9; MgSO_4_, 1.2; NaH_2_PO_4_, 1.2; CaCl_2_, 1.8; NaHCO_3_, 8.0; and Hepes, 20.0 (pH 7.4) exposing to 1% O_2_ for 1 h to balance the medium with a hypoxic atmosphere. Ischemia was stimulated by replacing the DMEM with the ischemic medium and maintaining the dishes under hypoxic gases (O_2_/N_2_/CO_2_, 1%/93%/5%). Cultured cardiac myocytes (H9c2 cells) were subjected to hypoxic condition by immediately replacing the medium with the hypoxic medium. After incubating in the hypoxic condition for the indicated time, the cells were collected for an individual experiment.

### 4.3. Determination of Cardiomyocyte Mitochondrial Viability

The assessment of cell viability was a modification of the MTT assay. The assay was based upon the principle of reduction of MTT into blue formazan pigment in the viable cells. This reduction occurs only in the presence of intact cell membranes and functional mitochondria. MTT was dissolved in 0.005 g/mL phosphate-buffered saline (PBS). At the end of an experiment, 100 μL MTT was added to each well. Cells were subsequently incubated for 3 h at 37 °C in an atmosphere of 5% CO_2_. This time period was found to be optimal for color development associated with formazan product formation. After the incubation period, the medium was removed. Then, 500 μL of isopropanol was added to each well and gently agitated for 10 min. This caused the cell membranes to lyse and liberate the formazan pigment. After standing for 10 min, the optical density (OD) was determined spectrophotometrically at 540 and 630 nm wavelength. Finally, the values were expressed as percentages of the control values.

### 4.4. Morphological Assessment and Quantification of Apoptotic H9c2 Cardiomyoblast

Hoechst 33342, a fluorescent nuclear binding dye, which allows a clear distinction between apoptotic and normal cells on the basis of nuclear morphology (chromatin condensation and fragmentation) was used to quantify the apoptotic myocytes. Hoechst 33342 (prepared in PBS) was added to the culture medium to a final concentration of 10 μg/mL. Cells were evaluated by fluorescence microscopy according to the following grading system: normal nuclei (blue chromatin with organized structure) and apoptotic cells (bright fluorescent chromatin which is highly condensed or fragmented). For each experimental condition, three separate cell populations were prepared.

### 4.5. DNA Ladder

DNA was isolated from H9c2 cells using the DNA isolation kit (Gentra Puregene Tissue Kit, Waltham, Massachusetts, USA) according to the manufacturer’s instructions. After precipitation by ethanol, DNA was dissolved in hydration solution. DNA (30 μg) was pipetted onto a 1.8% agarose gel and was stained with 0.5 μg/mL ethidium bromide. DNA fragments in the gel were visualized under ultraviolet (UV) light.

### 4.6. Measurement of Intracellular and Mitochondrial Calcium Concentration

The cells were sedimented, resuspended in the HBSS, and then incubated for 40 min with 5 μM fura-2-acetoxymethyl ester. Fura-2-loaded cells were washed twice with the HBSS and resuspended in the B2 buffer. Fluorescence emission at 510 nm wavelength from two excitation wavelengths (340 nm and 380 nm) were measured every 0.1 s (RF-5301PC, SHIMADZU, Kyoto, Japan). The ratio of fluorescence intensities from the two wavelengths was monitored as an estimate of intracellular calcium concentration [Ca^2+^]i.

Mitochondrial calcium ion concentration was estimated from Rhod-2 fluorescence. Cardiac cells grown on coverslips were exposed to Rhod-2-AM (2 μM), dissolved in PBS, at 37 °C for 30 min. Rhod-2 fluorescence, representing mitochondrial calcium, was elicited by excitation at 540 nm and emission was measured at a wavelength of 590 nm. To test whether the dye was specifically localized to mitochondria, we used fluorescence microscopy (Zeiss, Berlin, Germany).

### 4.7. Fluorescent Measurement of Intracellular Reactive Oxygen Species (ROS)

Intracellular oxidant stress was monitored by measuring the changes in fluorescence resulting from intracellular probe oxidation as previously described [17,40]. The probe 2’,7’-dichlorodihydrofluorescein diacetate/DCFH-DA (Molecular Probes, Eugene, OR, USA) diffuses across cell membranes. Subsequently, it is hydrolyzed by intracellular esterases to the polar and nonfluorescent DCFH form that is retained intracellularly. Subsequent oxidation by ROS, particularly H_2_O_2_ and hydroxyl radical, yield the fluorescent product of DCF. Thus, the increases in DCFH oxidation to DCF (i.e., increases in DCF fluorescence) are suggestive of H_2_O_2_ or hydroxyl generation. Cells were loaded with 10 μM DCFH-DA and incubated for 20 min at 37 °C, and analyzed by Partec CyFlow with excitation and emission settings of 495 nm and 525 nm, respectively.

### 4.8. Measurement of Nitrite Release

Nitrite (NO_2_^−^) content was used as an index of nitric oxide (NO) production. Nitrite level was determined in the supernatant of cell cultures by spectrophotometry, with Griess reagent (Promega, Madison, Wis.). An amount of 150 μL of the sample was added to 150 μL of freshly prepared Griess reagent in a 96-well plate, and the absorbance was read at 550 nm. The nitrite concentration was calculated with a sodium nitrite standard.

### 4.9. Immunoblot Analysis

Cultures were harvested in ice-cold lysis buffer. Equal amounts of protein from cleared lysates were fractionated in 7.5–12% SDS-polyacrylamide gels and then underwent electro-blotting in polyvinylidene plus difluoride membrane (Millipore, Burlington, Massachusetts, USA). Blots were stained with Ponceau Red to monitor the transfer of proteins. Membranes were blocked for 1 h at room temperature with 5% non-fat milk in TBS (25 mM Tris, 137 mM NaCl) containing 0.05% Tween-20 and incubated with the respective primary antibodies for 2–4 h in the milk. After washing, the blots were incubated for 1 h with horseradish peroxidase-conjugated secondary antibody and visualized using enhanced chemiluminescence kits (ECL, Amersham Pharmacia Biotech, St. Louis, Missouri, USA).

### 4.10. Statistical Analysis

Data are expressed as mean ± SEM. Statistical comparisons were performed by one-way analysis of variance and Newman–Keul’s hoc test for all treatment-versus-control and treatment-versus-treatment comparisons. The difference was considered significant at *p* < 0.05.

## 5. Conclusions

Overall, our data indicate that KMUP-1 has powerful protective effects against hypoxia-induced apoptosis in H9c2 cells. The mechanisms appear to involve an apoptosis prevention that is facilitated by the restoration of calcium influx and intracellular ROS generation in myocardial ischemia, further activating the NO-cGMP-MAPK signaling pathways. KMUP-1 treatment may be a promising clinical approach for ischemic heart disease. Further investigations are needed to achieve the beneficial application of KMUP-1.

## Figures and Tables

**Table 1:** Effect of KMUP-1 on plasma nitric oxide concentration (µM).

Normoxic Control	Hypoxia + KMUP-1			
	0 μM	0.1 μM	1 μM	10 μM
1.84 ± 0.39	3.94 ± 0.07	4.29 ± 0.19 **	4.56 ± 0.14 ^#^	5.26 ± 0.13 ^##^

All values were expressed as mean ± SEM, *n* = 3. ** *p* < 0.01 vs. normoxia, ^#^ *p* < 0.05 vs. hypoxia, ^##^ *p* < 0.01 vs. hypoxia.
